# Supplementary material for: Nothing else matters? Tree diameter and living status have more effects than biogeoclimatic context on microhabitat number and occurrence: An analysis in French forest reserves
Source: PLoS One. 2019 May 9;14(5):e0216500. doi: 10.1371/journal.pone.0216500 (PMC6508731; doi:10.1371/journal.pone.0216500)
Supplement: S1 Table — (DOCX) [file pone.0216500.s002.docx]

**Supporting Information**

S1 Table: Distribution of plots and trees across the study sites (see map, Figure 1)

| Site | Number of plots | Number of trees |
| --- | --- | --- |
| Artoise | 59 | 322 |
| Assan | 82 | 633 |
| Aulp du Seuil | 41 | 553 |
| Bannes | 143 | 630 |
| Beaux Monts | 81 | 525 |
| Bourg d’Oisans Vieille Morte | 7 | 10 |
| Butte de Malvran | 37 | 154 |
| Chaume Charlemagne | 65 | 623 |
| Citeaux | 51 | 755 |
| Col du Coq | 31 | 222 |
| Combe d’Ire | 91 | 629 |
| Dame Blanche | 56 | 277 |
| Dunes et Marais d’Hourtin | 40 | 147 |
| Foret du Langenberg | 39 | 394 |
| Foret Irreguliere de la Petite Pierre Sud | 178 | 1493 |
| Glaciere | 44 | 734 |
| Grand Tanargue | 39 | 580 |
| Grands Monts | 52 | 281 |
| Griffe au Diable | 38 | 124 |
| Haut Tuileau | 100 | 599 |
| Haute chaine du Jura | 137 | 1686 |
| Hautes Vosges | 114 | 1149 |
| Hengstberg | 67 | 231 |
| Ile Falcon | 1 | 2 |
| Ilots Cevennes | 44 | 482 |
| Lutzelhardt | 32 | 46 |
| Marais de Lavours | 8 | 24 |
| Montaigu | 95 | 998 |
| Nonnenthal | 62 | 462 |
| Partias | 2 | 4 |
| Plateau de Combe Noire | 65 | 550 |
| Quinquendolle | 86 | 431 |
| Ravin de Valbois | 104 | 447 |
| Roc de Chere | 57 | 1180 |
| Sources de l’Ardeche | 28 | 232 |
| Tanet Gazon du Faing | 54 | 854 |
| Tetes d’Alpe | 95 | 1132 |
| Tourbiere des Charmes | 19 | 289 |
| Tourbiere des Dauges | 27 | 176 |
| Valat de l’Hort de Dieu | 35 | 224 |
| Vercors | 279 | 976 |
| Vernay | 62 | 851 |
| Verrieres | 36 | 196 |
