# Supplementary material for: Nothing else matters? Tree diameter and living status have more effects than biogeoclimatic context on microhabitat number and occurrence: An analysis in French forest reserves
Source: PLoS One. 2019 May 9;14(5):e0216500. doi: 10.1371/journal.pone.0216500 (PMC6508731; doi:10.1371/journal.pone.0216500)
Supplement: S2 Table — DBH: Diameter at Breast Height; SE: standard error of the mean: p = p value; ***p<0.001; **p<0.01; *p<0.05. Beech: Fagus sylvatica; fir: Abies alba; oak: Quercus spp.; pine: Pinus spp.; and spruce: Picea abies. (DOCX) [file pone.0216500.s003.docx]

S2 Table: Scaled estimates for number of microhabitat types per tree from a generalised linear mixed model with a Poisson error distribution and plot nested in site as a random effect. DBH: Diameter at Breast Height; SE: standard error of the mean: p = p value; ***p<0.001; **p<0.01; *p<0.05. Beech: Fagus sylvatica; fir: Abies alba; oak: Quercus spp.; pine: Pinus spp.; and spruce: Picea abies.

| Parameter | Estimate | SE | p |  |
| --- | --- | --- | --- | --- |
| Intercept | 0.8198 | 0.0954 | <0.001 | *** |
| DBH | 0.2265 | 0.0359 | <0.001 | *** |
| Fir | -0.3196 | 0.0482 | <0.001 | *** |
| Oak | 0.2060 | 0.0502 | <0.001 | *** |
| Pine | -0.4070 | 0.0838 | <0.001 | *** |
| Spruce | -0.4386 | 0.0558 | <0.001 | *** |
| Living status (Living trees) | -0.3004 | 0.0338 | <0.001 | *** |
| pH | -0.0170 | 0.0506 | 0.7372 | ns |
| Elevation | 0.1136 | 0.0380 | 0.0028 | ** |
| DBH:Fir | -0.0576 | 0.0469 | 0.2193 | ns |
| DBH:Oak | -0.0112 | 0.0474 | 0.8128 | ns |
| DBH:Pine | -0.1282 | 0.0886 | 0.1478 | ns |
| DBH:Spruce | -0.0525 | 0.0541 | 0.3318 | ns |
| DBH: Living status (Living trees) | -0.0098 | 0.0368 | 0.7894 | ns |
| DBH:pH | 0.0460 | 0.0077 | <0.001 | *** |
| Living status (Living trees):pH | -0.0508 | 0.0189 | 0.0072 | ** |
| Fir:pH | -0.0362 | 0.0248 | 0.1445 | ns |
| Oak:pH | 0.0537 | 0.0221 | 0.0153 | * |
| Pine:pH | 0.0976 | 0.0350 | 0.0053 | ** |
| Spruce:pH | -0.0311 | 0.0273 | 0.2553 | ns |
| Fir:Living status (Living trees) | 0.0455 | 0.0491 | 0.3534 | ns |
| Oak:Living status (Living trees) | -0.1354 | 0.0500 | 0.0068 | ** |
| Pine:Living status (Living trees) | 0.0017 | 0.0840 | 0.9837 | ns |
| Spruce:Living status (Living trees) | 0.0708 | 0.0574 | 0.2173 | ns |
| DBH:Fir:Living status (Living trees) | -0.1034 | 0.0491 | 0.0352 | * |
| DBH:Oak:Living status (Living trees) | -0.0160 | 0.0484 | 0.7409 | ns |
| DBH:Pine:Living status (Living trees) | 0.1964 | 0.0954 | 0.0396 | * |
| DBH:Spruce:Living status (Living trees) | -0.1154 | 0.0572 | 0.0435 | * |
