# Supplementary material for: Nothing else matters? Tree diameter and living status have more effects than biogeoclimatic context on microhabitat number and occurrence: An analysis in French forest reserves
Source: PLoS One. 2019 May 9;14(5):e0216500. doi: 10.1371/journal.pone.0216500 (PMC6508731; doi:10.1371/journal.pone.0216500)
Supplement: S3 Table — Beech: Fagus sylvatica; fir: Abies alba; oak: Quercus spp.; pine: Pinus spp.; and spruce: Picea abies. (DOCX) [file pone.0216500.s004.docx]

S3 Table: Accumulation levels of microhabitats per tree (number of microhabitats and occurrence) for a Diameter at Breast Height (DBH) increment from 50 cm to 100 cm issued from generalised linear mixed models with Poisson (number) and binomial (occurrence) error distributions. Beech: Fagus sylvatica; fir: Abies alba; oak: Quercus spp.; pine: Pinus spp.; and spruce: Picea abies.

|  | Living trees | | | | |  | Dead trees | | | | |
| --- | --- | --- | --- | --- | --- | --- | --- | --- | --- | --- | --- |
| Microhabitats | Beech | Fir | Oak | Pine | Spruce |  | Beech | Fir | Oak | Pine | Spruce |
| All | 1.173 | 0.261 | 0.999 | 1.641 | 0.212 |  | 1.608 | 0.919 | 1.802 | 0.732 | 1.006 |
| Base cavities | 0.176 | 0.064 | 0.052 | 0.427 | 0.026 |  | 0.345 | 0.063 | 0.061 | -0.004 | 0.181 |
| Trunk cavities | 0.077 | 0.013 | 0.036 | 0.026 | 0.05 |  | 0.346 | 0.058 | 0.282 | 0.005 | 0.055 |
| Canopy cavities | 0.018 | 0.002 | 0.005 | 0.000 | 0.000 |  | 0.000 | 0.062 | 0.002 | 0.000 | 0.011 |
| Woodp. cavities | 0.005 | 0.001 | 0.004 | 0.209 | 0.006 |  | 0.033 | 0.027 | 0.007 | 0.131 | 0.023 |
| Cracks | 0.028 | 0.001 | 0.057 | 0.007 | 0.012 |  | 0.077 | -0.004 | 0.01 | -0.001 | -0.003 |
| Woodp. feeding holes | 0.032 | 0.005 | 0.024 | 0.002 | 0.004 |  | 0.417 | 0.305 | 0.411 | 0.022 | 0.233 |
| Rot | 0.011 | 0.003 | 0.033 | 0.042 | 0.001 |  | 0.234 | 0.007 | 0.267 | 0.039 | -0.019 |
| Injuries | 0.043 | -0.003 | 0.043 | 0.012 | -0.004 |  | -0.005 | -0.003 | 0.006 | 0.012 | -0.003 |
| Conks of fungi | 0.047 | 0.022 | 0.003 | 0.002 | -0.001 |  | 0.150 | 0.240 | 0.143 | -0.01 | 0.270 |
| Bark characteristics | 0.004 | 0.002 | 0.013 | 0.003 | 0.000 |  | 0.021 | 0.09 | 0.121 | 0.014 | -0.028 |
| Moss cover >50% | 0.196 | 0.243 | -0.032 | -0.071 | 0.246 |  | 0.086 | 0.161 | -0.001 | 0.626 | 0.277 |
| Lichen cover > 50% | 0.063 | 0.097 | 0.017 | 0.174 | -0.045 |  | -0.017 | 0.02 | 0.005 | -0.027 | 0.026 |
| Ivy cover >50% | 0.001 | 0.002 | 0.058 | -0.002 | 0.006 |  | 0.001 | 0.000 | 0.003 | 0.011 | 0.000 |
| Small branches | 0.200 | 0.348 | 0.214 | 0.147 | 0.559 |  | -0.003 | 0.049 | -0.015 | -0.008 | 0.007 |
| Medium branches | 0.498 | 0.36 | 0.526 | 0.324 | 0.019 |  | 0.015 | 0.010 | 0.007 | -0.007 | 0.013 |
| Large branches | 0.049 | 0.003 | 0.012 | 0.081 | 0.000 |  | 0.021 | 0.006 | 0.000 | 0.011 | 0.000 |
| Crown skeleton | 0.000 | 0.001 | 0.000 | 0.090 | 0.005 |  | -0.001 | 0.000 | 0.000 | 0.124 | -0.006 |
| Forks | 0.279 | 0.050 | 0.309 | 0.152 | -0.012 |  | -0.001 | 0.049 | 0.001 | -0.001 | 0.048 |
| Broken stem | 0.012 | -0.021 | -0.003 | -0.017 | -0.01 |  | 0.116 | 0.024 | 0.052 | -0.025 | 0.052 |
