# Supplementary material for: Nothing else matters? Tree diameter and living status have more effects than biogeoclimatic context on microhabitat number and occurrence: An analysis in French forest reserves
Source: PLoS One. 2019 May 9;14(5):e0216500. doi: 10.1371/journal.pone.0216500 (PMC6508731; doi:10.1371/journal.pone.0216500)
Supplement: S4 Table — DBH: Diameter at Breast Height; SE: standard error of the mean. p = p value; ***p<0.001; **p<0.01; *p<0.05. (DOCX) [file pone.0216500.s005.docx]

S4 Table: Scaled estimates for number of microhabitat types per tree for beech (Fagus sylvatica) and pine (Pinus spp.) from a generalised linear mixed model with a Poisson error distribution and plot nested in site as a random effect. DBH: Diameter at Breast Height; SE: standard error of the mean. p = p value; ***p<0.001; **p<0.01; *p<0.05.

|  | Beech | | | | Pine | | | |
| --- | --- | --- | --- | --- | --- | --- | --- | --- |
|  | Estimate | SE | p |  | Estimate | SE | p |  |
| Intercept | 0.759 | 0.097 | <0.001 | *** | 0.285 | 0.186 | 0.126 | ns |
| DBH | 0.147 | 0.034 | <0.001 | *** | 0.101 | 0.046 | 0.030 | * |
| Living status (Living trees) | -0.270 | 0.033 | <0.001 | *** | -0.441 | 0.069 | <0.001 | *** |
| pH | 0.032 | 0.064 | 0.614 | ns | 0.173 | 0.190 | 0.363 | ns |
| Elevation | -0.030 | 0.060 | 0.617 | ns | -0.250 | 0.168 | 0.137 | ns |
| DBH:Living status (Living trees) | 0.030 | 0.034 | 0.375 | ns | 0.124 | 0.051 | 0.016 | * |
| DBH:pH | 0.045 | 0.010 | <0.001 | *** | 0.169 | 0.067 | 0.012 | * |
| DBH:Elevation | -0.007 | 0.009 | 0.477 | ns | -0.105 | 0.064 | 0.097 | (*) |
| Living status (Living trees):pH | -0.078 | 0.033 | 0.017 | * | -0.171 | 0.155 | 0.269 | ns |
| Living status (Living trees):Elevation | 0.120 | 0.036 | 0.001 | ** | 0.302 | 0.166 | 0.068 | (*) |
