# Supplementary material for: Nothing else matters? Tree diameter and living status have more effects than biogeoclimatic context on microhabitat number and occurrence: An analysis in French forest reserves
Source: PLoS One. 2019 May 9;14(5):e0216500. doi: 10.1371/journal.pone.0216500 (PMC6508731; doi:10.1371/journal.pone.0216500)
Supplement: S5 Table — DBH: Diameter at Breast Height; SE: standard error of the mean. Beech: Fagus sylvatica; fir: Abies alba; oak: Quercus spp.; pine: Pinus spp.; and spruce: Picea abies. p = p value; ***p<0.001; **p<0.01; *p<0.05. (DOCX) [file pone.0216500.s006.docx]

S5 Table: Scaled estimates for occurrence of microhabitat types per tree from a generalised linear mixed model with a binomial error distribution and plot nested in site as a random effect. DBH: Diameter at Breast Height; SE: standard error of the mean. Beech: Fagus sylvatica; fir: Abies alba; oak: Quercus spp.; pine: Pinus spp.; and spruce: Picea abies. p = p value; ***p<0.001; **p<0.01; *p<0.05.

|  | Base cavities | | | | | | | Trunk cavities | | | | | | | | Canopy cavities | | | | | | | | Woodpecker cavities | | | | | | | | Cracks | | | | | | | | | |
| --- | --- | --- | --- | --- | --- | --- | --- | --- | --- | --- | --- | --- | --- | --- | --- | --- | --- | --- | --- | --- | --- | --- | --- | --- | --- | --- | --- | --- | --- | --- | --- | --- | --- | --- | --- | --- | --- | --- | --- | --- | --- |
|  | Estimate | | SE | | p | |  | Estimate | | | SE | | p | |  | Estimate | | | SE | | p | |  | Estimate | | | SE | | p | |  | Estimate | | | SE | | p | |  | | |
| Intercept | | | -2.81 | | 0.353 | | <0.001 | | | *** | -2.562 | | 0.266 | | <0.001 | | | *** | -7.499 | | 1.281 | | <0.001 | | | *** | -3.513 | | 0.324 | | <0.001 | | | *** | -3.306 | | 0.324 | | <0.001 | | *** |
| DBH | | | 0.85 | | 0.207 | | <0.001 | | | *** | 0.824 | | 0.189 | | <0.001 | | | *** | -0.007 | | 1.375 | | 0.996 | | | ns | 0.388 | | 0.257 | | 0.131 | | | ns | 0.449 | | 0.217 | | 0.039 | | * |
| Fir | | | -0.99 | | 0.344 | | 0.004 | | | ** | -1.319 | | 0.304 | | <0.001 | | | *** | -1.223 | | 2.008 | | 0.543 | | | ns | -1.651 | | 0.525 | | 0.002 | | | ** | -1.275 | | 0.366 | | <0.001 | | *** |
| Oak | | | -1.077 | | 0.367 | | 0.003 | | | ** | -0.409 | | 0.322 | | 0.203 | | | ns | 1.389 | | 1.239 | | 0.262 | | | ns | -0.477 | | 0.453 | | 0.292 | | | ns | 0.102 | | 0.373 | | 0.784 | | ns |
| Pine | | | -2.019 | | 0.655 | | 0.002 | | | ** | -1.256 | | 0.519 | | 0.016 | | | * | -11.291 | | 16.5 | | 0.494 | | | ns | -0.669 | | 0.674 | | 0.321 | | | ns | -3.909 | | 0.879 | | <0.001 | | *** |
| Spruce | | | -1.357 | | 0.343 | | <0.001 | | | *** | -2.06 | | 0.419 | | <0.001 | | | *** | -1.502 | | 2.208 | | 0.496 | | | ns | -2.253 | | 0.647 | | <0.001 | | | *** | -0.842 | | 0.4 | | 0.035 | | * |
| Living status (Living trees) | | | -0.395 | | 0.206 | | 0.056 | | | (*) | -0.926 | | 0.183 | | <0.001 | | | *** | 0.967 | | 1.21 | | 0.424 | | | ns | -2.11 | | 0.281 | | <0.001 | | | *** | -0.94 | | 0.215 | | <0.001 | | *** |
| pH | | | -0.266 | | 0.286 | | 0.351 | | | ns | -0.256 | | 0.225 | | 0.256 | | | ns | 0.969 | | 0.756 | | 0.2 | | | ns | 0.256 | | 0.261 | | 0.326 | | | ns | 0.125 | | 0.267 | | 0.638 | | ns |
| Elevation | | | 0.255 | | 0.219 | | 0.244 | | | ns | 0.641 | | 0.176 | | <0.001 | | | *** | -0.011 | | 0.354 | | 0.976 | | | ns | -0.032 | | 0.19 | | 0.867 | | | ns | 0.174 | | 0.191 | | 0.361 | | ns |
| DBH:Fir | | | -0.35 | | 0.32 | | 0.275 | | | ns | -0.372 | | 0.28 | | 0.184 | | | ns | 2.131 | | 1.571 | | 0.175 | | | ns | 0.31 | | 0.429 | | 0.47 | | | ns | -0.641 | | 0.392 | | 0.102 | | ns |
| DBH:Oak | | | -0.298 | | 0.304 | | 0.327 | | | ns | 0.125 | | 0.265 | | 0.636 | | | ns | 0.663 | | 1.419 | | 0.64 | | | ns | -0.226 | | 0.388 | | 0.56 | | | ns | -0.306 | | 0.321 | | 0.341 | | ns |
| DBH:Pine | | | -0.956 | | 0.625 | | 0.126 | | | ns | -0.682 | | 0.529 | | 0.197 | | | ns | -9.992 | | 13.3 | | 0.454 | | | ns | 0.019 | | 0.607 | | 0.975 | | | ns | -1.206 | | 0.745 | | 0.105 | | ns |
| DBH:Spruce | | | 0.103 | | 0.321 | | 0.747 | | | ns | -0.259 | | 0.355 | | 0.467 | | | ns | 1.65 | | 1.579 | | 0.296 | | | ns | 0.326 | | 0.43 | | 0.448 | | | ns | -0.524 | | 0.358 | | 0.143 | | ns |
| DBH:Living status (Living trees) | | | -0.188 | | 0.211 | | 0.372 | | | ns | -0.373 | | 0.195 | | 0.055 | | | (*) | 0.915 | | 1.378 | | 0.507 | | | ns | 0.115 | | 0.27 | | 0.668 | | | ns | -0.028 | | 0.222 | | 0.9 | | ns |
| DBH:pH | | | -0.009 | | 0.054 | | 0.867 | | | ns | 0.044 | | 0.062 | | 0.48 | | | ns | -0.146 | | 0.163 | | 0.372 | | | ns | 0.129 | | 0.105 | | 0.222 | | | ns | -0.061 | | 0.068 | | 0.368 | | ns |
| Living status (Living trees):pH | | | -0.227 | | 0.359 | | 0.526 | | | ns | -0.881 | | 0.352 | | 0.012 | | | * | -0.757 | | 2.151 | | 0.725 | | | ns | 1.266 | | 0.573 | | 0.027 | | | * | 0.054 | | 0.403 | | 0.893 | | ns |
| Fir:pH | | | 0.301 | | 0.363 | | 0.407 | | | ns | -0.187 | | 0.322 | | 0.561 | | | ns | -1.167 | | 1.231 | | 0.343 | | | ns | 0.559 | | 0.468 | | 0.232 | | | ns | -1.461 | | 0.393 | | <0.001 | | *** |
| Oak: pH | | | -1.039 | | 0.644 | | 0.107 | | | ns | -1.285 | | 0.571 | | 0.024 | | | * | 10.847 | | 16.4 | | 0.511 | | | ns | 0.59 | | 0.674 | | 0.381 | | | ns | 2.538 | | 0.748 | | 0.001 | | ** |
| Pine: pH | | | 1.094 | | 0.348 | | 0.002 | | | ** | -1.735 | | 0.616 | | 0.005 | | | ** | -10.626 | | 108.9 | | 0.922 | | | ns | 1.569 | | 0.649 | | 0.016 | | | * | -0.291 | | 0.451 | | 0.519 | | ns |
| Spruce: pH | | | 0.149 | | 0.142 | | 0.293 | | | ns | 0.25 | | 0.126 | | 0.048 | | | * | -0.821 | | 0.657 | | 0.211 | | | ns | -0.044 | | 0.196 | | 0.822 | | | ns | -0.434 | | 0.152 | | 0.004 | | ** |
| Fir:Living status (Living trees) | | | 0.07 | | 0.186 | | 0.706 | | | ns | -0.597 | | 0.207 | | 0.004 | | | ** | 0.601 | | 1.028 | | 0.559 | | | ns | -0.165 | | 0.318 | | 0.603 | | | ns | 0.071 | | 0.219 | | 0.747 | | ns |
| Oak:Living status (Living trees) | | | 0.375 | | 0.177 | | 0.034 | | | * | -0.241 | | 0.187 | | 0.199 | | | ns | -1.067 | | 0.404 | | 0.008 | | | ** | -0.345 | | 0.279 | | 0.216 | | | ns | 0.053 | | 0.261 | | 0.838 | | ns |
| Pine:Living status (Living trees) | | | 0.915 | | 0.525 | | 0.081 | | | (*) | 0.229 | | 0.41 | | 0.577 | | | ns | 0.058 | | 0.9 | | 0.949 | | | ns | 0.214 | | 0.387 | | 0.581 | | | ns | 1.484 | | 0.509 | | 0.004 | | ** |
| Spruce:Living status (Living trees) | | | -0.018 | | 0.147 | | 0.902 | | | ns | -0.376 | | 0.297 | | 0.206 | | | ns | -1.108 | | 2.206 | | 0.616 | | | ns | 0.295 | | 0.518 | | 0.569 | | | ns | 0.111 | | 0.215 | | 0.604 | | ns |
| DBH:Fir:Living status (Living trees) | | | 0.28 | | 0.334 | | 0.403 | | | ns | 0.418 | | 0.32 | | 0.192 | | | ns | -2.472 | | 1.693 | | 0.144 | | | ns | -0.655 | | 0.477 | | 0.17 | | | ns | 0.301 | | 0.42 | | 0.474 | | ns |
| DBH:Oak:Living status (Living trees) | | | 0.155 | | 0.308 | | 0.614 | | | ns | -0.021 | | 0.272 | | 0.938 | | | ns | -0.78 | | 1.423 | | 0.584 | | | ns | 0.226 | | 0.405 | | 0.578 | | | ns | 0.578 | | 0.331 | | 0.08 | | (*) |
| DBH:Pine:Living status (Living trees) | | | 1.572 | | 0.684 | | 0.021 | | | * | 1.091 | | 0.625 | | 0.081 | | | (*) | 9.922 | | 13.357 | | 0.458 | | | ns | 0.398 | | 0.666 | | 0.55 | | | ns | 1.482 | | 0.747 | | 0.047 | | * |
| DBH:Spruce:Living status (Living trees) | | | -0.295 | | 0.333 | | 0.375 | | | ns | 0.833 | | 0.438 | | 0.057 | | | (*) | -3.628 | | 128.023 | | 0.977 | | | ns | -0.085 | | 0.463 | | 0.854 | | | ns | 0.49 | | 0.394 | | 0.214 | | ns |

S5 Table (continued)

|  | Woodpecker feeding holes | | | | Rot | | | | Injuries | | | | Conks of fungi | | | | Bark characteristics | | | | |
| --- | --- | --- | --- | --- | --- | --- | --- | --- | --- | --- | --- | --- | --- | --- | --- | --- | --- | --- | --- | --- | --- |
|  | Estimate | SE | p |  | Estimate | SE | p |  | Estimate | SE | p |  | Estimate | SE | p |  | | Estimate | SE | p |  |
| Intercept | -0.92 | 0.303 | 0.002 | ** | -3.213 | 0.348 | <0.001 | *** | -4.215 | 0.404 | <0.001 | *** | -0.534 | 0.231 | 0.021 | * | | -2.726 | 0.368 | <0.001 | *** |
| DBH | 0.805 | 0.19 | <0.001 | *** | 0.928 | 0.207 | <0.001 | *** | -0.236 | 0.342 | 0.49 | ns | 0.224 | 0.15 | 0.135 | ns | | 0.22 | 0.197 | 0.263 | ns |
| Fir | 0.352 | 0.251 | 0.161 | ns | -1.126 | 0.327 | 0.001 | ** | -0.961 | 0.445 | 0.031 | * | -0.453 | 0.23 | 0.048 | * | | -0.238 | 0.342 | 0.487 | ns |
| Oak | 0.353 | 0.274 | 0.196 | ns | 1.385 | 0.321 | <0.001 | *** | -0.258 | 0.529 | 0.626 | ns | -1.959 | 0.261 | <0.001 | *** | | 1.69 | 0.295 | <0.001 | *** |
| Pine | -0.98 | 0.392 | 0.012 | * | -1.083 | 0.64 | 0.091 | (*) | -1.298 | 0.54 | 0.016 | * | -2.174 | 0.563 | <0.001 | *** | | -0.094 | 0.525 | 0.857 | ns |
| Spruce | -0.573 | 0.274 | 0.037 | * | -0.389 | 0.333 | 0.242 | ns | -1.099 | 0.595 | 0.065 | (*) | -1.195 | 0.283 | <0.001 | *** | | 0.595 | 0.333 | 0.074 | (*) |
| Living status (Living trees) | -4.721 | 0.23 | <0.001 | *** | -1.018 | 0.223 | <0.001 | *** | 1.701 | 0.304 | <0.001 | *** | -4.368 | 0.184 | <0.001 | *** | | -3.257 | 0.222 | <0.001 | *** |
| pH | -0.107 | 0.277 | 0.699 | ns | 0.523 | 0.285 | 0.067 | (*) | 0.649 | 0.249 | 0.009 | ** | -0.643 | 0.205 | 0.002 | ** | | 0.673 | 0.31 | 0.03 | * |
| Elevation | -0.447 | 0.207 | 0.031 | * | 0.249 | 0.218 | 0.255 | ns | -0.175 | 0.145 | 0.229 | ns | -0.512 | 0.179 | 0.004 | ** | | -0.41 | 0.277 | 0.138 | ns |
| DBH:Fir | -0.281 | 0.23 | 0.221 | ns | -0.79 | 0.288 | 0.006 | ** | -0.395 | 0.531 | 0.457 | ns | 0.203 | 0.191 | 0.287 | ns | | 0.224 | 0.278 | 0.421 | ns |
| DBH:Oak | 0.227 | 0.275 | 0.409 | ns | -0.348 | 0.27 | 0.198 | ns | 0.402 | 0.489 | 0.412 | ns | 0.14 | 0.222 | 0.529 | ns | | -0.001 | 0.268 | 0.998 | ns |
| DBH:Pine | -0.825 | 0.368 | 0.025 | * | -0.384 | 0.559 | 0.492 | ns | 0.418 | 0.544 | 0.442 | ns | -0.364 | 0.633 | 0.565 | ns | | -0.098 | 0.48 | 0.838 | ns |
| DBH:Spruce | -0.383 | 0.261 | 0.142 | ns | -1.395 | 0.332 | <0.001 | *** | -0.69 | 0.734 | 0.347 | ns | 0.424 | 0.25 | 0.09 | (*) | | -0.493 | 0.299 | 0.099 | (*) |
| DBH:Living status (Living trees) | <0.001 | 0.21 | 0.999 | ns | -0.609 | 0.212 | 0.004 | ** | 0.52 | 0.343 | 0.13 | ns | 0.367 | 0.165 | 0.026 | * | | 0.228 | 0.209 | 0.276 | ns |
| DBH:pH | 0.162 | 0.072 | 0.024 | * | 0.075 | 0.065 | 0.251 | ns | 0.076 | 0.034 | 0.028 | * | -0.042 | 0.063 | 0.508 | ns | | 0.136 | 0.088 | 0.122 | ns |
| Living status (Living trees):pH | -0.665 | 0.365 | 0.069 | (*) | 0.559 | 0.339 | 0.099 | (*) | 0.722 | 0.446 | 0.106 | ns | -0.564 | 0.357 | 0.114 | ns | | -0.271 | 0.405 | 0.504 | ns |
| Fir:pH | 0.445 | 0.3 | 0.137 | ns | -2.106 | 0.329 | <0.001 | *** | -0.196 | 0.529 | 0.711 | ns | 1.636 | 0.28 | <0.001 | *** | | -1.975 | 0.312 | <0.001 | *** |
| Oak: pH | 0.786 | 0.451 | 0.081 | (*) | -1.32 | 0.7 | 0.059 | (*) | 0.248 | 0.523 | 0.636 | ns | 0.809 | 0.89 | 0.363 | ns | | -0.951 | 0.755 | 0.208 | ns |
| Pine: pH | -0.037 | 0.366 | 0.92 | ns | -1.219 | 0.404 | 0.003 | ** | 1.253 | 0.601 | 0.037 | * | 0.269 | 0.45 | 0.55 | ns | | -1.72 | 0.489 | <0.001 | *** |
| Spruce: pH | 0.356 | 0.141 | 0.011 | * | -0.386 | 0.15 | 0.01 | * | -0.412 | 0.146 | 0.005 | ** | 0.223 | 0.121 | 0.066 | (*) | | -0.983 | 0.154 | <0.001 | *** |
| Fir:Living status (Living trees) | -0.016 | 0.227 | 0.944 | ns | -0.356 | 0.18 | 0.048 | * | -0.565 | 0.093 | <0.001 | *** | 0.513 | 0.188 | 0.006 | ** | | 0.814 | 0.321 | 0.011 | * |
| Oak:Living status (Living trees) | 0.174 | 0.219 | 0.428 | ns | -0.305 | 0.231 | 0.187 | ns | -0.276 | 0.116 | 0.018 | * | 0.275 | 0.207 | 0.184 | ns | | -0.259 | 0.221 | 0.242 | ns |
| Pine:Living status (Living trees) | -0.204 | 0.29 | 0.482 | ns | -0.466 | 0.43 | 0.278 | ns | -0.645 | 0.157 | <0.001 | *** | 0.444 | 0.361 | 0.219 | ns | | 0.288 | 0.386 | 0.455 | ns |
| Spruce:Living status (Living trees) | 0.005 | 0.277 | 0.986 | ns | -0.833 | 0.227 | <0.001 | *** | -1.081 | 0.119 | <0.001 | *** | 0.164 | 0.229 | 0.474 | ns | | 0.193 | 0.307 | 0.53 | ns |
| DBH:Fir:Living status (Living trees) | -0.232 | 0.313 | 0.459 | ns | 0.737 | 0.3 | 0.014 | * | 0.03 | 0.535 | 0.956 | ns | -0.226 | 0.273 | 0.407 | ns | | -0.458 | 0.338 | 0.175 | ns |
| DBH:Oak:Living status (Living trees) | -0.529 | 0.29 | 0.068 | (*) | 0.312 | 0.282 | 0.269 | ns | -0.409 | 0.492 | 0.406 | ns | -0.44 | 0.24 | 0.066 | (*) | | -0.204 | 0.287 | 0.477 | ns |
| DBH:Pine:Living status (Living trees) | 0.562 | 0.478 | 0.24 | ns | 0.967 | 0.727 | 0.184 | ns | -0.671 | 0.554 | 0.226 | ns | 0.464 | 1.214 | 0.702 | ns | | 0.048 | 1.001 | 0.962 | ns |
| DBH:Spruce:Living status (Living trees) | 0.058 | 0.334 | 0.861 | ns | 1.1 | 0.379 | 0.004 | ** | 0.213 | 0.739 | 0.773 | ns | -1.409 | 0.45 | 0.002 | ** | | -0.036 | 0.448 | 0.936 | ns |

S5 Table (continued)

|  | Moss (>50%) | | | | Lichen (>50%) | | | | Ivy (>50%) | | | | Small branches | | | | Medium branches | | | |
| --- | --- | --- | --- | --- | --- | --- | --- | --- | --- | --- | --- | --- | --- | --- | --- | --- | --- | --- | --- | --- |
|  | Estimate | SE | p |  | Estimate | SE | p |  | Estimate | SE | p |  | Estimate | SE | p |  | Estimate | SE | p |  |
| Intercept | -0.168 | 0.618 | 0.785 | ns | -3.5 | 0.776 | <0.001 | *** | -6.781 | 0.655 | <0.001 | *** | -4.219 | 0.429 | <0.001 | *** | -4.441 | 0.353 | <0.001 | *** |
| DBH | 0.174 | 0.189 | 0.358 | ns | -0.333 | 0.27 | 0.218 | ns | 0.29 | 0.407 | 0.476 | ns | -0.212 | 0.35 | 0.544 | ns | 0.359 | 0.351 | 0.306 | ns |
| Fir | -1.535 | 0.256 | <0.001 | *** | 0.189 | 0.318 | 0.552 | ns | -0.889 | 0.621 | 0.152 | ns | 0.774 | 0.356 | 0.03 | * | 1.531 | 0.341 | <0.001 | *** |
| Oak | -1.071 | 0.287 | <0.001 | *** | 0.975 | 0.427 | 0.022 | * | 1.084 | 0.512 | 0.034 | * | 0.333 | 0.432 | 0.441 | ns | 1.344 | 0.363 | <0.001 | *** |
| Pine | -1.973 | 0.529 | <0.001 | *** | 0.334 | 0.479 | 0.485 | ns | 0.63 | 0.972 | 0.517 | ns | 0.776 | 0.505 | 0.124 | ns | 1.17 | 0.518 | 0.024 | * |
| Spruce | -2.116 | 0.305 | <0.001 | *** | -0.98 | 0.339 | 0.004 | ** | 0.36 | 0.568 | 0.526 | ns | 0.341 | 0.46 | 0.459 | ns | -1.892 | 1.329 | 0.155 | ns |
| Living status (Living trees) | 0.832 | 0.173 | <0.001 | *** | 1.519 | 0.237 | <0.001 | *** | 0.524 | 0.487 | 0.283 | ns | 2.509 | 0.291 | <0.001 | *** | 1.382 | 0.279 | <0.001 | *** |
| pH | 0.09 | 0.391 | 0.819 | ns | -0.496 | 0.453 | 0.274 | ns | 1.524 | 0.492 | 0.002 | ** | 0.417 | 0.253 | 0.099 | (*) | 0.481 | 0.224 | 0.032 | * |
| Elevation | -0.785 | 0.29 | 0.007 | ** | 2.353 | 0.388 | <0.001 | *** | -2.124 | 0.351 | <0.001 | *** | -0.241 | 0.158 | 0.127 | ns | -0.237 | 0.159 | 0.136 | ns |
| DBH:Fir | 0.276 | 0.273 | 0.312 | ns | 0.421 | 0.343 | 0.219 | ns | -0.046 | 0.482 | 0.925 | ns | 0.615 | 0.393 | 0.118 | ns | -0.276 | 0.406 | 0.496 | ns |
| DBH:Oak | -0.165 | 0.276 | 0.55 | ns | 0.494 | 0.463 | 0.286 | ns | -0.237 | 0.449 | 0.598 | ns | -0.822 | 0.507 | 0.105 | ns | -0.308 | 0.419 | 0.462 | ns |
| DBH:Pine | 1.284 | 0.672 | 0.056 | (*) | 0.242 | 0.523 | 0.643 | ns | 0.593 | 1.215 | 0.625 | ns | -0.892 | 0.542 | 0.1 | ns | -0.459 | 0.581 | 0.43 | ns |
| DBH:Spruce | 0.586 | 0.302 | 0.053 | (*) | 0.505 | 0.371 | 0.173 | ns | -0.437 | 0.534 | 0.413 | ns | 0.393 | 0.458 | 0.391 | ns | 0.444 | 0.799 | 0.578 | ns |
| DBH:Living status (Living trees) | 0.362 | 0.193 | 0.061 | (*) | 0.578 | 0.273 | 0.034 | * | -0.041 | 0.41 | 0.921 | ns | 0.537 | 0.351 | 0.127 | ns | 0.558 | 0.354 | 0.115 | ns |
| DBH:pH | 0.074 | 0.042 | 0.077 | (*) | 0.046 | 0.048 | 0.34 | ns | -0.001 | 0.091 | 0.991 | ns | -0.162 | 0.032 | <0.001 | *** | -0.022 | 0.043 | 0.608 | ns |
| Living status (Living trees):pH | 0.21 | 0.254 | 0.407 | ns | 0.499 | 0.317 | 0.115 | ns | 0.692 | 0.555 | 0.212 | ns | -1.262 | 0.354 | <0.001 | *** | -3.727 | 0.385 | <0.001 | *** |
| Fir:pH | 1.853 | 0.287 | <0.001 | *** | -0.852 | 0.422 | 0.043 | * | -0.433 | 0.501 | 0.388 | ns | 0.614 | 0.432 | 0.155 | ns | 0.131 | 0.361 | 0.718 | ns |
| Oak: pH | -2.15 | 0.549 | <0.001 | *** | -0.799 | 0.485 | 0.1 | ns | -1.047 | 1.018 | 0.304 | ns | 0.407 | 0.496 | 0.412 | ns | -0.239 | 0.508 | 0.639 | ns |
| Pine: pH | -0.963 | 0.305 | 0.002 | ** | 0.537 | 0.331 | 0.104 | ns | 0.117 | 0.539 | 0.828 | ns | -1.459 | 0.472 | 0.002 | ** | -0.546 | 1.373 | 0.691 | ns |
| Spruce: pH | -0.029 | 0.11 | 0.791 | ns | -0.383 | 0.126 | 0.002 | ** | -0.523 | 0.342 | 0.127 | ns | 0.005 | 0.108 | 0.967 | ns | -0.209 | 0.125 | 0.094 | (*) |
| Fir:Living status (Living trees) | 0.377 | 0.125 | 0.003 | ** | -0.718 | 0.161 | <0.001 | *** | 0.802 | 0.506 | 0.113 | ns | -0.752 | 0.103 | <0.001 | *** | -0.765 | 0.163 | <0.001 | *** |
| Oak:Living status (Living trees) | 0.172 | 0.141 | 0.224 | ns | -0.428 | 0.168 | 0.011 | * | 0.488 | 0.196 | 0.013 | * | -0.074 | 0.088 | 0.402 | ns | -0.256 | 0.095 | 0.007 | ** |
| Pine:Living status (Living trees) | 0.555 | 0.217 | 0.01 | * | -0.043 | 0.188 | 0.819 | ns | -0.065 | 0.397 | 0.87 | ns | -0.302 | 0.139 | 0.03 | * | 0.011 | 0.176 | 0.95 | ns |
| Spruce:Living status (Living trees) | 0.594 | 0.158 | <0.001 | *** | 0.105 | 0.166 | 0.525 | ns | 0.568 | 0.539 | 0.292 | ns | 0.147 | 0.132 | 0.268 | ns | -0.511 | 0.258 | 0.047 | * |
| DBH:Fir:Living status (Living trees) | -0.426 | 0.284 | 0.134 | ns | -0.483 | 0.351 | 0.169 | ns | 0.272 | 0.496 | 0.584 | ns | -0.201 | 0.398 | 0.614 | ns | 0.456 | 0.423 | 0.282 | ns |
| DBH:Oak:Living status (Living trees) | -0.42 | 0.28 | 0.134 | ns | -0.531 | 0.467 | 0.255 | ns | 0.262 | 0.454 | 0.564 | ns | 0.859 | 0.508 | 0.091 | (*) | 0.327 | 0.422 | 0.439 | ns |
| DBH:Pine:Living status (Living trees) | -2.209 | 0.739 | 0.003 | ** | -0.173 | 0.568 | 0.761 | ns | -1.185 | 1.304 | 0.363 | ns | 1.251 | 0.552 | 0.023 | * | 0.964 | 0.597 | 0.106 | ns |
| DBH:Spruce:Living status (Living trees) | -0.704 | 0.321 | 0.028 | * | -0.895 | 0.386 | 0.02 | * | 0.696 | 0.549 | 0.205 | ns | 0.348 | 0.471 | 0.461 | ns | -0.337 | 0.828 | 0.684 | ns |

S5 Table (continued)

|  | Large branches | | | | Crown skeleton | | | | Forks | | | | Broken stems | | | |
| --- | --- | --- | --- | --- | --- | --- | --- | --- | --- | --- | --- | --- | --- | --- | --- | --- |
|  | Estimate | SE | p |  | Estimate | SE | p |  | Estimate | SE | p |  | Estimate | SE | p |  |
| Intercept | -5.839 | 0.644 | <0.001 | *** | -5.822 | 0.933 | <0.001 | *** | -6.104 | 0.804 | <0.001 | *** | -3.511 | 0.361 | <0.001 | *** |
| DBH | 0.729 | 0.499 | 0.144 | ns | 0.12 | 0.304 | 0.694 | ns | -1.374 | 0.763 | 0.072 | (*) | 0.779 | 0.238 | 0.001 | ** |
| Fir | -1.928 | 1.504 | 0.2 | ns | -1.784 | 1.031 | 0.084 | . | 0.395 | 0.775 | 0.61 | ns | 0.121 | 0.31 | 0.696 | ns |
| Oak | -0.396 | 0.857 | 0.644 | ns | 0.091 | 0.424 | 0.83 | ns | 2.225 | 0.847 | 0.009 | ** | -0.347 | 0.44 | 0.43 | ns |
| Pine | 1.042 | 0.924 | 0.26 | ns | 0.732 | 0.637 | 0.25 | ns | 0.301 | 1.077 | 0.78 | ns | -0.867 | 0.532 | 0.103 | ns |
| Spruce | -18.493 | 3608.741 | 0.996 | ns | 1.755 | 0.502 | <0.001 | *** | 0.474 | 0.81 | 0.559 | ns | -0.331 | 0.355 | 0.351 | ns |
| Living status (Living trees) | -0.703 | 0.593 | 0.236 | ns | -4.754 | 0.512 | <0.001 | *** | 3.597 | 0.731 | <0.001 | *** | -0.25 | 0.266 | 0.347 | ns |
| pH | 0.032 | 0.403 | 0.937 | ns | -0.394 | 0.634 | 0.534 | ns | -0.209 | 0.299 | 0.485 | ns | 0.688 | 0.251 | 0.006 | ** |
| Elevation | -0.214 | 0.277 | 0.438 | ns | -0.784 | 0.304 | 0.01 | * | 1.543 | 0.198 | <0.001 | *** | 0.278 | 0.165 | 0.092 | (*) |
| DBH:Fir | 0.314 | 0.947 | 0.74 | ns | -0.539 | 0.839 | 0.52 | ns | 2.116 | 0.8 | 0.008 | ** | -0.551 | 0.286 | 0.054 | (*) |
| DBH:Oak | -0.683 | 0.742 | 0.357 | ns | -0.05 | 0.349 | 0.887 | ns | 1.43 | 0.865 | 0.098 | (*) | -0.135 | 0.331 | 0.682 | ns |
| DBH:Pine | 0.768 | 0.872 | 0.378 | ns | 0.666 | 0.506 | 0.188 | ns | 1.042 | 1.111 | 0.348 | ns | -1.74 | 0.512 | 0.001 | ** |
| DBH:Spruce | -1.007 | 4451.258 | 1 | ns | -0.904 | 0.495 | 0.068 | (*) | 2 | 0.804 | 0.013 | * | -0.405 | 0.313 | 0.195 | ns |
| DBH:Living status (Living trees) | 0.505 | 0.51 | 0.322 | ns | -0.049 | 0.425 | 0.908 | ns | 2.028 | 0.764 | 0.008 | ** | -0.53 | 0.242 | 0.028 | * |
| DBH:pH | -0.086 | 0.112 | 0.443 | ns | 0.372 | 0.153 | 0.015 | * | 0.138 | 0.038 | <0.001 | *** | 0.09 | 0.047 | 0.056 | (*) |
| Living status (Living trees):pH | -0.211 | 1.74 | 0.903 | ns | 2.824 | 0.933 | 0.002 | ** | -1.749 | 0.777 | 0.024 | * | 0.242 | 0.314 | 0.442 | ns |
| Fir:pH | 1.881 | 0.863 | 0.029 | * | 0.409 | 0.576 | 0.478 | ns | -2.486 | 0.845 | 0.003 | ** | 0.282 | 0.44 | 0.521 | ns |
| Oak: pH | -0.516 | 0.938 | 0.582 | ns | 2.212 | 0.729 | 0.002 | ** | -1.196 | 1.077 | 0.267 | ns | 1.084 | 0.523 | 0.038 | * |
| Pine: pH | 11.885 | 3608.739 | 0.997 | ns | 1.664 | 0.669 | 0.013 | * | -1.927 | 0.813 | 0.018 | * | 0.451 | 0.364 | 0.216 | ns |
| Spruce: pH | -0.01 | 0.312 | 0.975 | ns | 0.372 | 0.266 | 0.161 | ns | -0.201 | 0.166 | 0.226 | ns | -0.361 | 0.111 | 0.001 | ** |
| Fir:Living status (Living trees) | 0.169 | 0.728 | 0.816 | ns | 1.987 | 1.066 | 0.062 | (*) | 0.682 | 0.102 | <0.001 | *** | -0.354 | 0.121 | 0.003 | ** |
| Oak:Living status (Living trees) | -0.234 | 0.278 | 0.4 | ns | 0.181 | 0.576 | 0.753 | ns | 0.05 | 0.136 | 0.712 | ns | 0.186 | 0.203 | 0.359 | ns |
| Pine:Living status (Living trees) | -0.338 | 0.442 | 0.445 | ns | 1.75 | 0.679 | 0.01 | * | 0.319 | 0.178 | 0.073 | (*) | -0.126 | 0.188 | 0.504 | ns |
| Spruce:Living status (Living trees) | 2.271 | 6.236 | 0.716 | ns | 0.845 | 0.475 | 0.075 | (*) | -0.295 | 0.138 | 0.032 | * | -0.325 | 0.17 | 0.056 | (*) |
| DBH:Fir:Living status (Living trees) | -0.726 | 1.054 | 0.491 | ns | 1.364 | 0.902 | 0.131 | ns | -2.664 | 0.803 | 0.001 | ** | -0.43 | 0.297 | 0.147 | ns |
| DBH:Oak:Living status (Living trees) | 0.353 | 0.75 | 0.638 | ns | 0.645 | 0.471 | 0.171 | ns | -1.516 | 0.866 | 0.08 | (*) | -0.246 | 0.342 | 0.471 | ns |
| DBH:Pine:Living status (Living trees) | -0.34 | 0.955 | 0.722 | ns | 0.31 | 0.567 | 0.584 | ns | -1.093 | 1.121 | 0.329 | ns | 0.753 | 0.532 | 0.157 | ns |
| DBH:Spruce:Living status (Living trees) | 2.124 | 4451.258 | 1 | ns | 1.561 | 0.615 | 0.011 | * | -2.744 | 0.808 | 0.001 | ** | -0.293 | 0.327 | 0.371 | ns |
