# Supplementary material for: Nothing else matters? Tree diameter and living status have more effects than biogeoclimatic context on microhabitat number and occurrence: An analysis in French forest reserves
Source: PLoS One. 2019 May 9;14(5):e0216500. doi: 10.1371/journal.pone.0216500 (PMC6508731; doi:10.1371/journal.pone.0216500)

S1 Figure: Relationship between total number of microhabitats (N microhabitats per tree) and Diameter at Breast Height (DBH, cm) by genus for living trees only. Lines represent estimates from generalized mixed effect models with a Poisson error distribution. Ribbons show the 95% confidence interval of the mean. For this representation, pH and elevation were held constant. Ash: Fraxinus excelsior; beech: Fagus sylvatica; chestnut: Castanea sativa; fir: Abies alba; hornbeam: Carpinus betulus; larch: Larix decidua; maple: Acer spp., oak: Quercus spp.; pine: Pinus spp.; poplar: Populus spp.; and spruce: Picea abies.


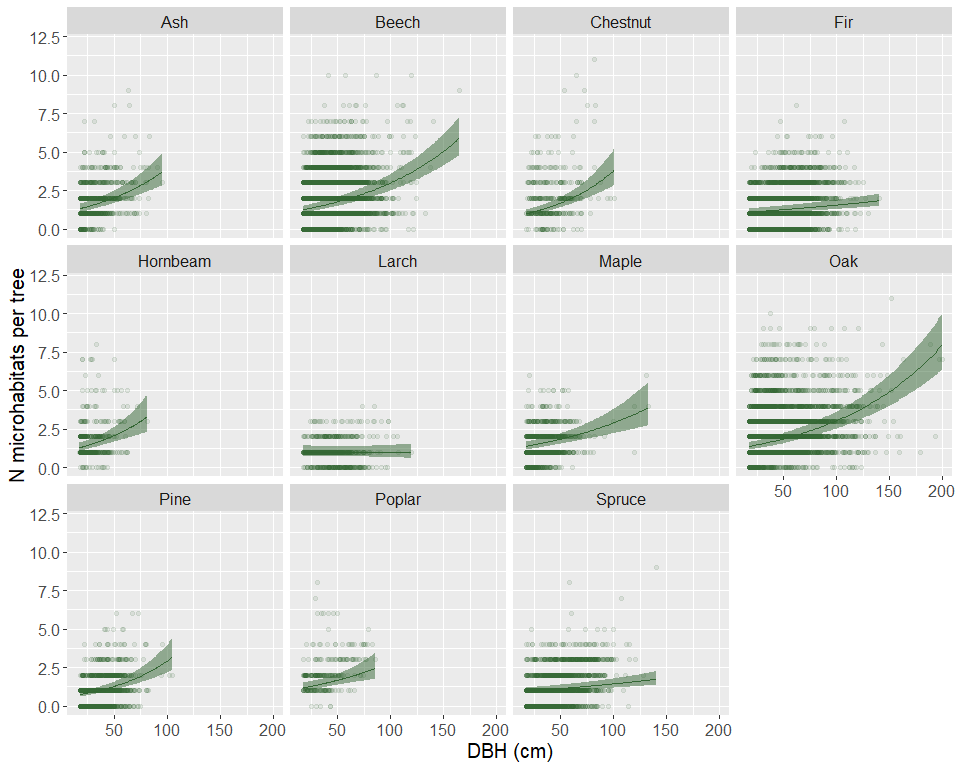

Supplement: S1 Fig — Ribbons show the 95% confidence interval of the mean. For this representation, pH and elevation were held constant. Ash: Fraxinus excelsior; beech: Fagus sylvatica; chestnut: Castanea sativa; fir: Abies alba; hornbeam: Carpinus betulus; larch: Larix decidua; maple: Acer spp., oak: Quercus spp.; pine: Pinus spp.; poplar: Populus spp.; and spruce: Picea abies. (DOCX) [file pone.0216500.s007.docx]
