# Supplementary material for: Nothing else matters? Tree diameter and living status have more effects than biogeoclimatic context on microhabitat number and occurrence: An analysis in French forest reserves
Source: PLoS One. 2019 May 9;14(5):e0216500. doi: 10.1371/journal.pone.0216500 (PMC6508731; doi:10.1371/journal.pone.0216500)

S2 Figure: Relationship between occurrence of microhabitats per tree and Diameter at Breast Height (DBH, cm) by species and living status (living vs. dead standing trees). Lines represent estimates from generalized mixed effect models with a binomial error distribution. Ribbons show the 95% confidence interval of the mean. For the representation, pH and elevation were held constant. Beech: Fagus sylvatica; fir: Abies alba; oak: Quercus spp.; pine: Pinus spp.; and spruce: Picea abies.


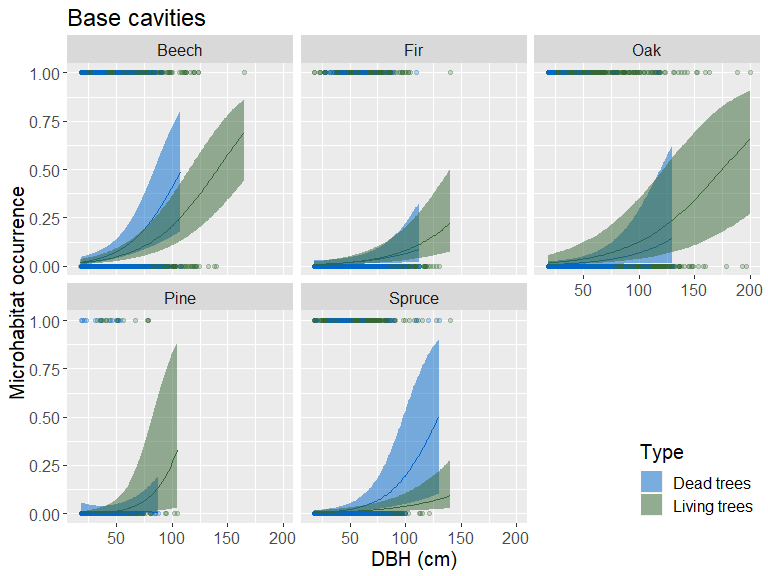


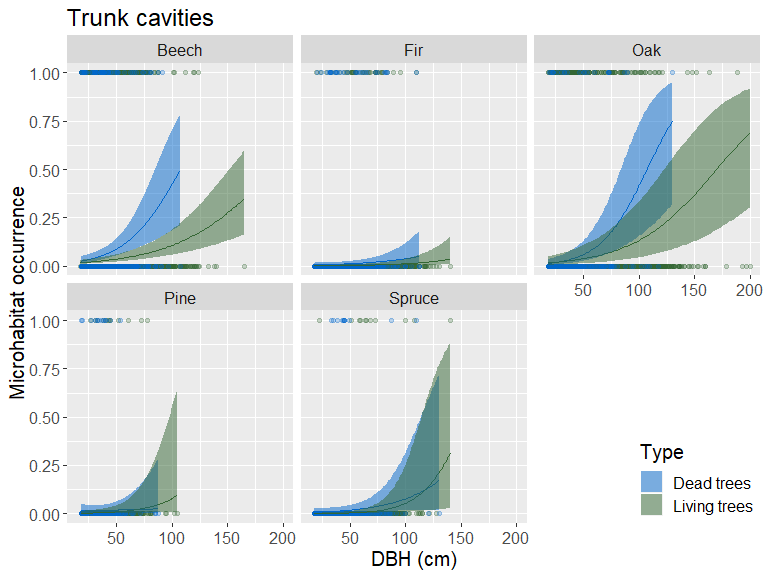


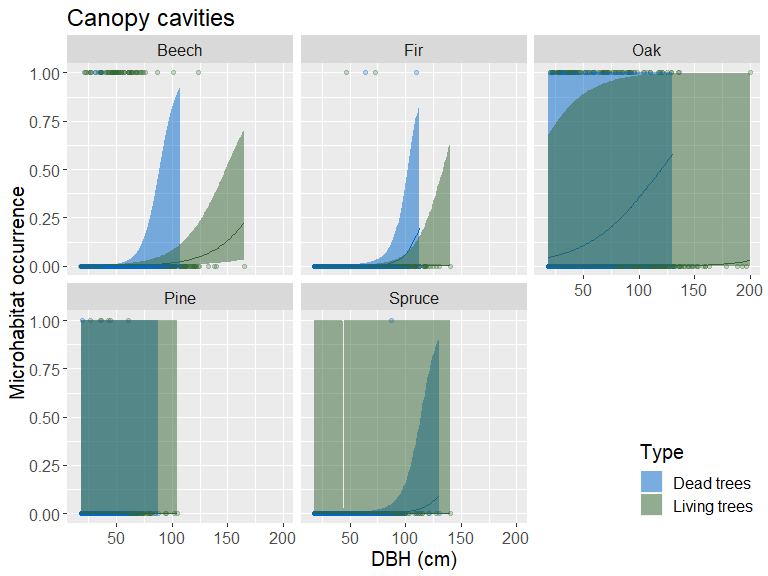


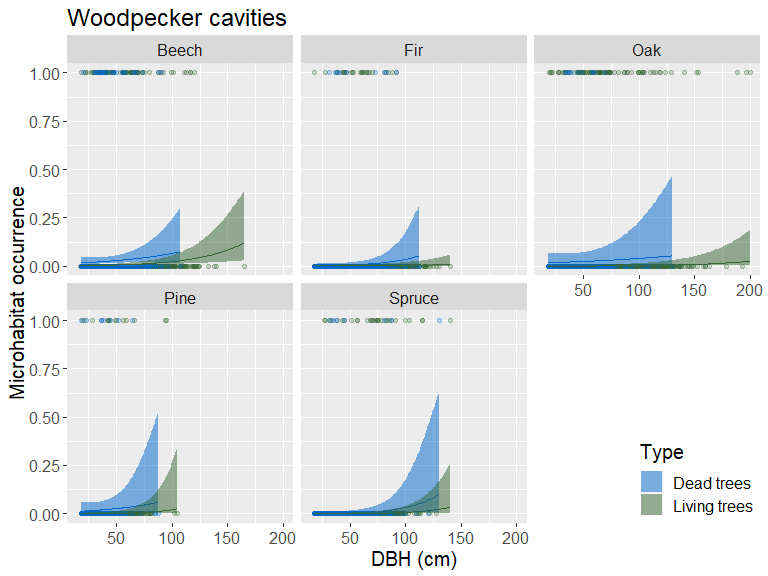


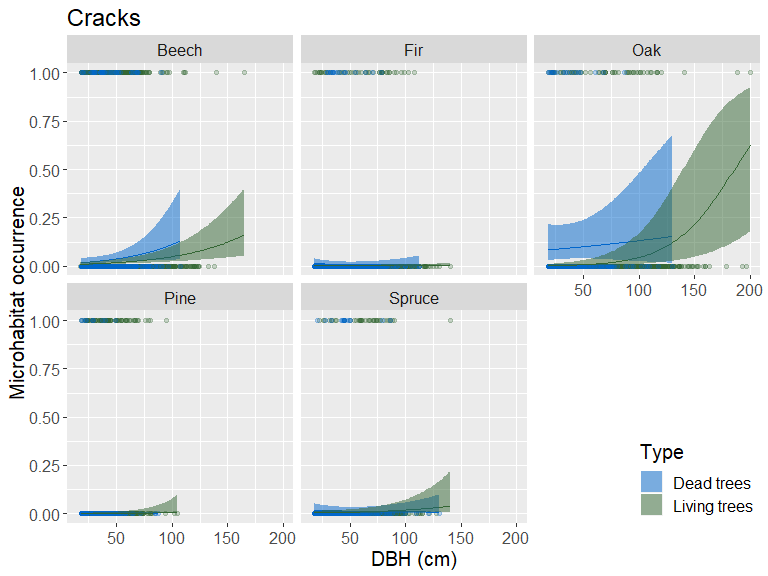


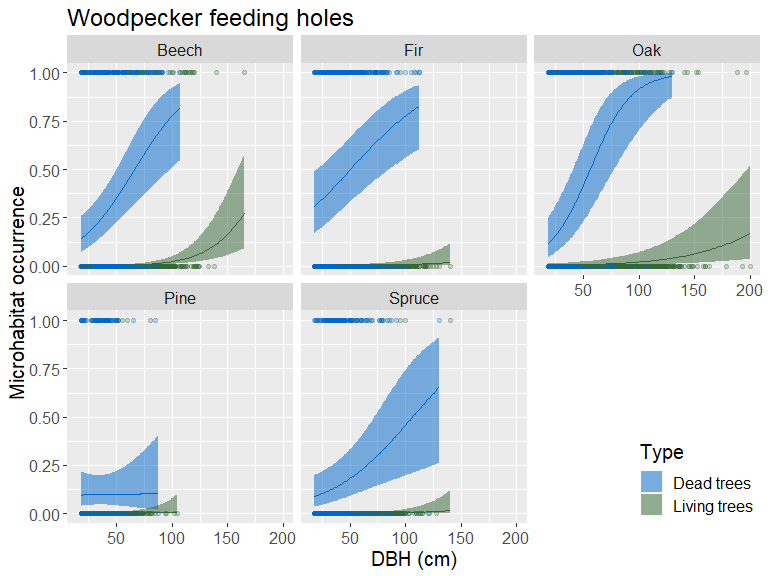


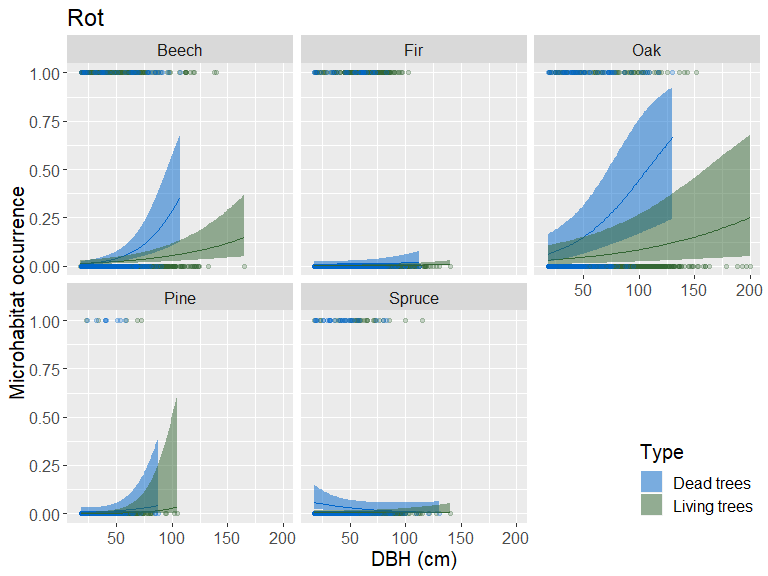


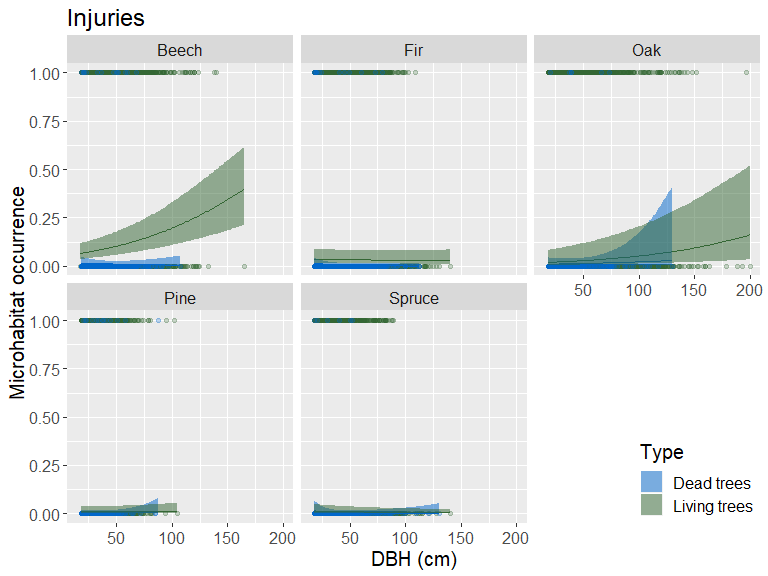


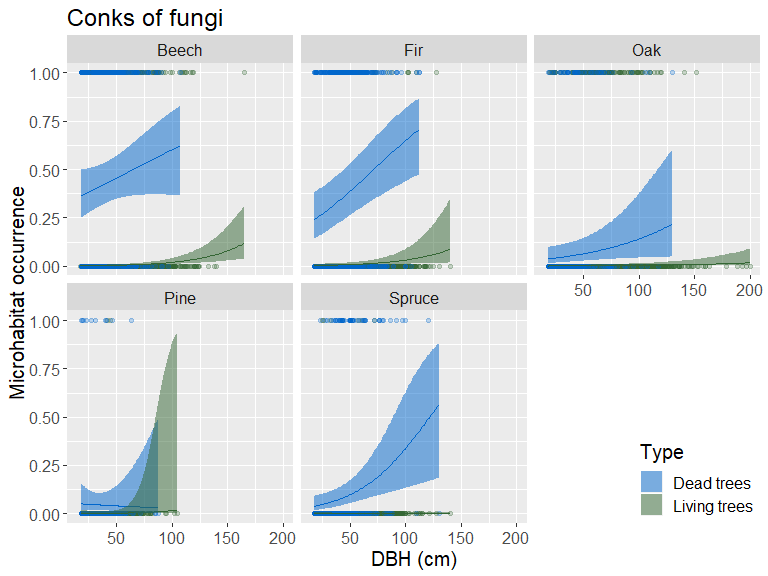


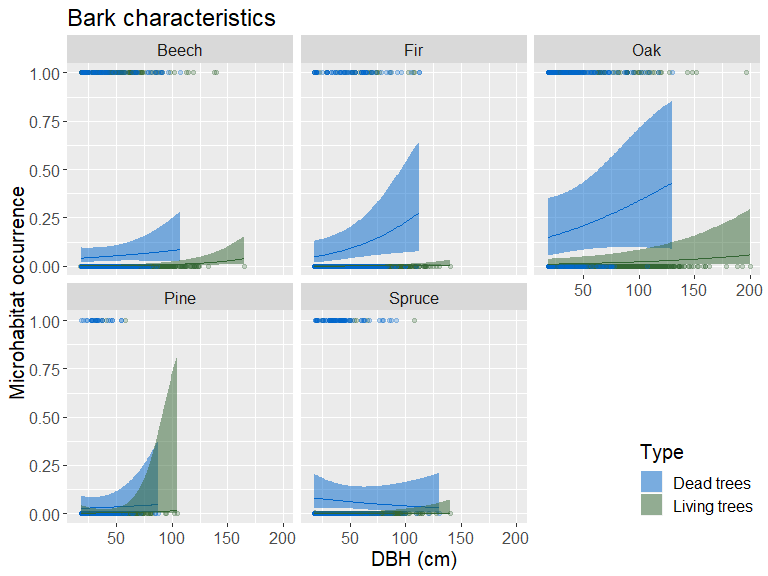


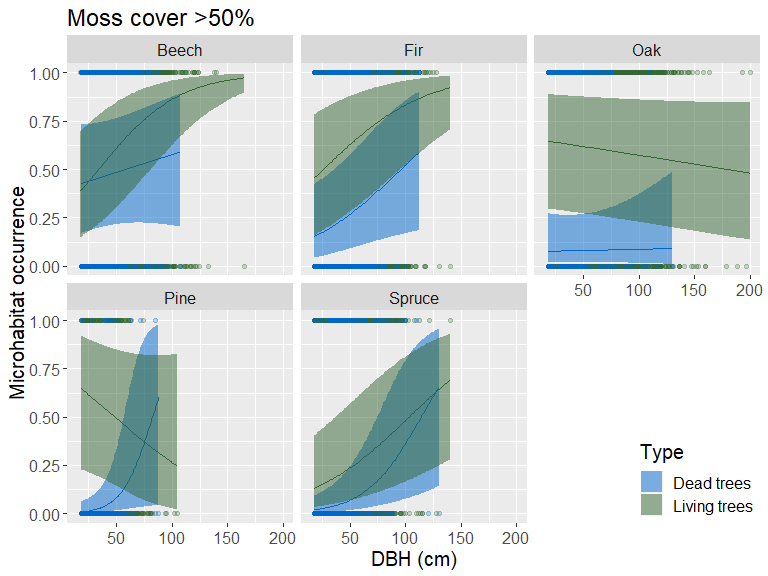


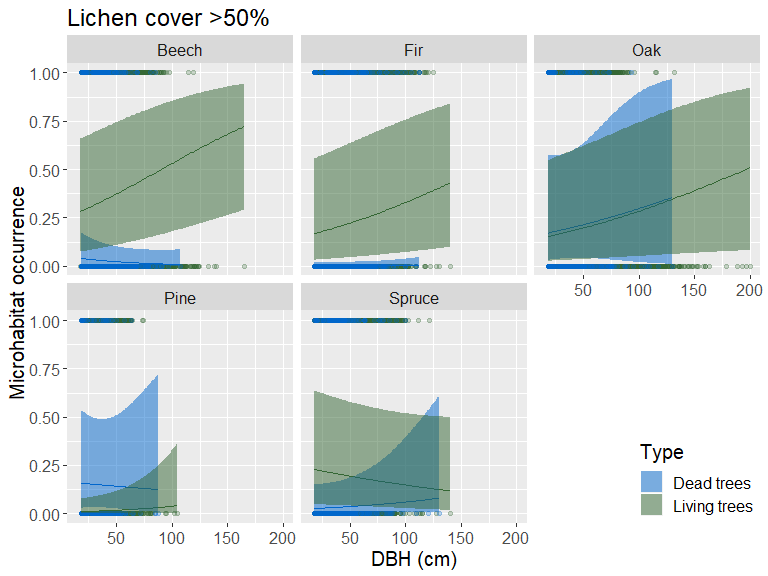

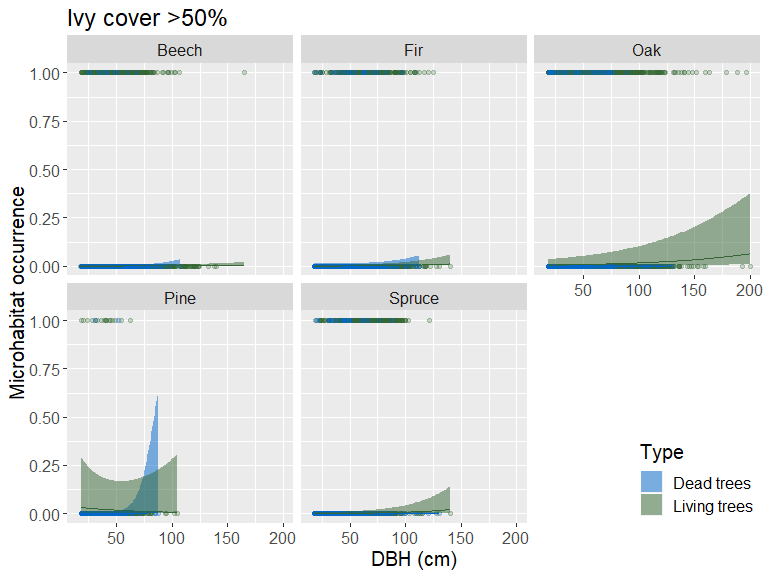


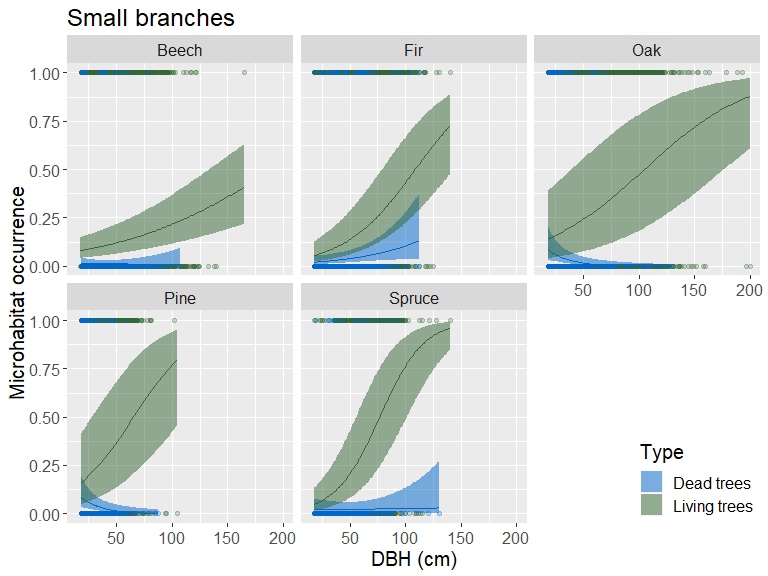


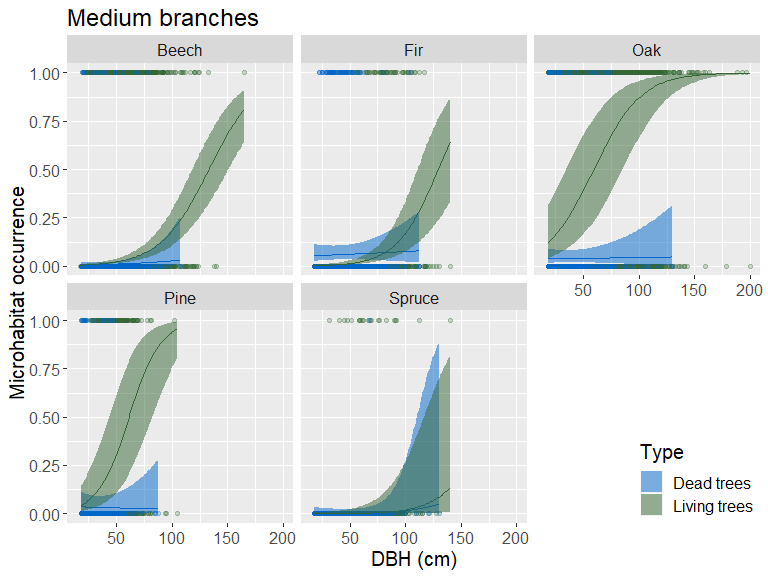


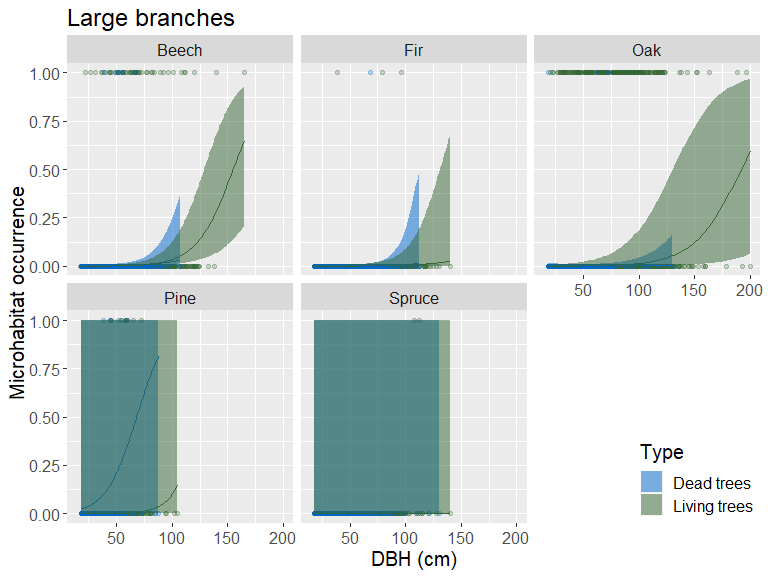


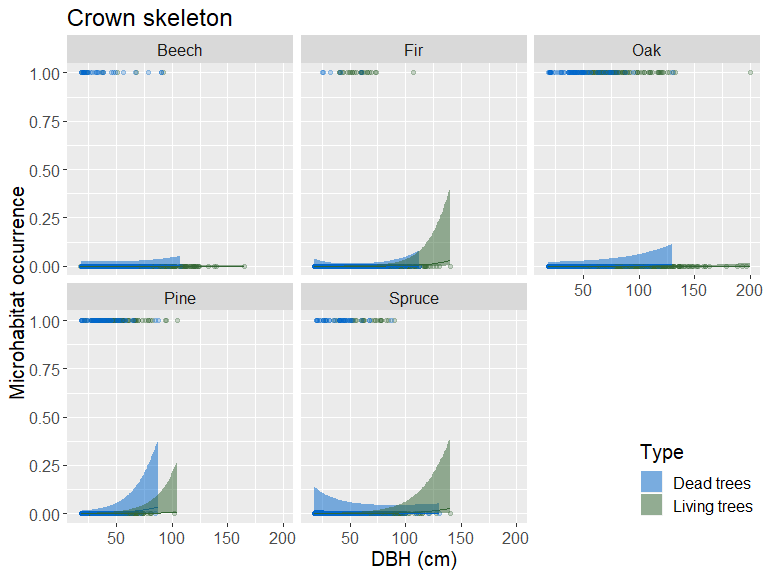


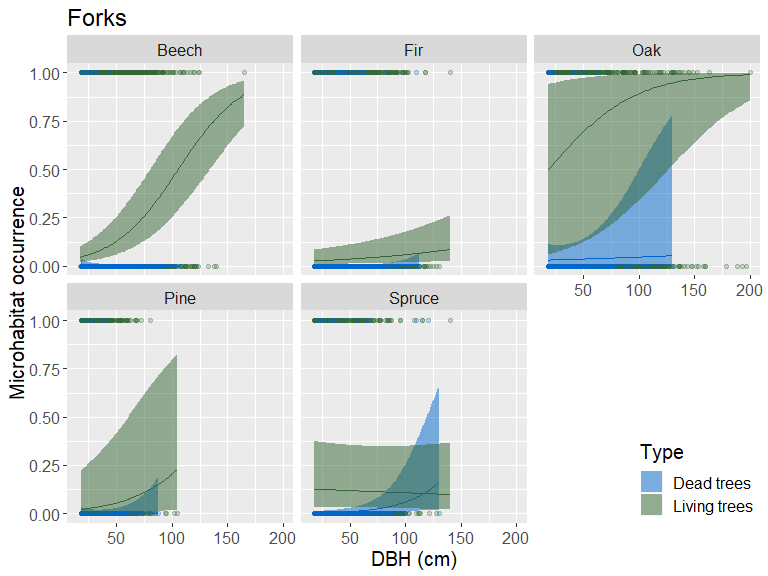


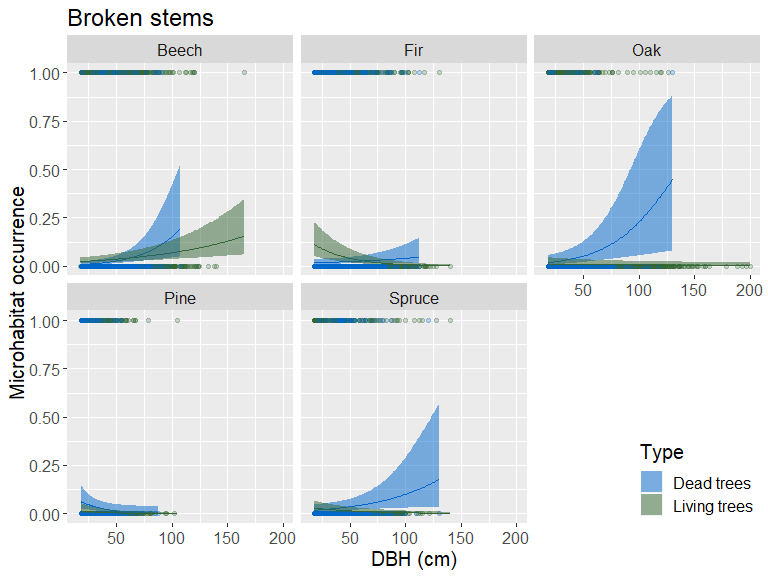

Supplement: S2 Fig — Lines represent estimates from generalized mixed effect models with a binomial error distribution. Ribbons show the 95% confidence interval of the mean. For the representation, pH and elevation were held constant. Beech: Fagus sylvatica; fir: Abies alba; oak: Quercus spp.; pine: Pinus spp.; and spruce: Picea abies. (DOCX) [file pone.0216500.s008.docx]
